# Silicon-Mediated Mitigation of Moderate Ammonium Stress in Maize Seedlings

**DOI:** 10.3390/plants14243793

**Published:** 2025-12-12

**Authors:** Hilário Júnior de Almeida, Anelisa de Aquino Vidal Lacerda Soares, Victor Manuel Vergara Carmona, Renato de Mello Prado

**Affiliations:** 1 Faculty of Agricultural and Veterinary Sciences, São Paulo State University (UNESP), Jaboticabal 14884-000, Brazil; rm.prado@fcav.unesp.br; 2 São Paulo Agency for Agribusiness Technology (APTA), Bauru 17030-000, Brazil; avidal@sp.gov.br; 3 Sociedad de Investigación y Servicios BioTECNOS Ltda., Camino a Pangal km 2.5, San Javier 3660000, Chile; vivergara66@gmail.com

**Keywords:** nitrato redutase, prolina, abiotic stress, plant growth, beneficial element

## Abstract

Intensive irrigated agriculture relies heavily on nitrogen fertilization, which may cause ammonium accumulation, highly detrimental to sensitive seedlings. Silicon application has emerged as a potential strategy to mitigate this stress, although the underlying mechanisms remain poorly understood. To evaluate this effect, maize seedlings were grown in nutrient solution under five N concentrations (1.4, 3.6, 7.1, 14.3, and 28.6 mmol L^−1^), applied in the presence or absence of silicon (1.8 mmol L^−1^ Si). The nitrogen source was a mixture of nitrate and ammonium in a N-NO_3_^−^: N-NH_4_^+^ ratio of 4:5. Silicon was supplied as monosilicic acid (H_2_SiO_3_). Plant growth, leaf area, root morphology (length, diameter, density), N and Si accumulation, uptake and utilization efficiency, SPAD index, nitrate reductase activity, and proline content were evaluated. Silicon supplementation enhanced nitrate reductase activity, SPAD values, leaf area, and root traits, reduced proline in roots and shoots, and improved N uptake and partitioning. Among the tested N concentrations, 14.3 mmol L^−1^ achieved the highest efficiency of nutrient absorption and biomass production, highlighting silicon as a sustainable strategy to mitigate ammonium stress in maize seedlings.

## 1. Introduction

Nitrogen (N) is an essential macronutrient for plant development, and its limitation can significantly compromise biomass production and crop yield. In agriculture, nitrogen fertilizers containing nitrate (NO_3_^−^) and ammonium (NH_4_^+^) are widely applied to meet plant nutritional demand [1,2]. Plants absorb both NO_3_^−^ and NH_4_^+^, whose uptake mechanisms and assimilation differ in energetic and biochemical aspects [3]. The assimilation of NH_4_^+^ is metabolically more efficient than that of NO_3_^−^, since NH_4_^+^ can be directly incorporated into organic compounds immediately after absorption, without the need for energy-dependent enzymatic reduction processes [4]. However, at high concentrations, NH_4_^+^ can induce stress in plants, as the C/N ratio and rhizosphere acidification contribute to this effect [5].

However, most plants exhibit growth and developmental inhibition when exposed to high concentrations of NH_4_^+^, particularly when it is the sole nitrogen source available [6]. Excessive NH_4_^+^ supply, generally at millimolar levels, can trigger multiple phytotoxic effects, including leaf chlorosis, inhibition of root and shoot growth, rhizosphere acidification, defects in protein glycosylation, accumulation of reactive oxygen species (ROS), cytoplasmic acidification, depletion of inorganic cations and organic acids, impairment of photosystem function, and disturbances in hormonal signaling [7,8]. This problem is especially relevant in intensive agricultural systems, where high nitrogen fertilizer inputs can lead to ammonium accumulation in the rhizosphere, resulting in substantial physiological stress to crops.

In this context, silicon (Si) stands out as the only element currently known to effectively alleviate both biotic and abiotic stresses in a wide range of plant species. Evidence indicates that Si acts as a beneficial element in mitigating stress, including caused by excess NH_4_^+^, through mechanisms such as enhancing nutrient use efficiency, increasing antioxidant system activity, elevating photosynthetic pigment content, and stimulating photosynthesis [9,10,11,12]. For instance, in rice plants exposed to high N concentrations, Si modulated the expression of nitrogen-uptake-related genes, leading to their downregulation and consequently limiting nutrient absorption [13]. In wheat, Si supplementation enhanced nitrate reductase (NR) activity in response to increasing NH_4_^+^ concentrations in the nutrient solution [14]. Similar responses have been reported in maize [15], yellow passion fruit seedlings [16], *Eucalyptus* spp. [17], and cotton [18].

In addition to these effects, the accumulation of compatible organic solutes, such as proline, constitutes one of the physiological and biochemical mechanisms adopted by plants to tolerate biotic and abiotic stresses, including those resulting from stress caused by excess N in the form of NH_4_^+^. Proline plays a crucial role in nitrogen assimilation [19], being an essential constituent of various plant biomolecules and a key factor influencing productivity across agricultural systems [20]. Under stress conditions, proline levels in plants can increase up to 100-fold compared with non-stressed conditions [21]. In the presence of Si, increases in photosystem II quantum efficiency, reductions in cellular electrolyte leakage, and decreases in free proline levels have been observed, all contributing to enhanced stress tolerance and resulting in improved plant growth and biomass accumulation [22].

Despite evidence that Si can mitigate ammonium stress in several plant species, a gap still exists regarding the physiological mechanisms underlying this response in maize seedlings, especially under conditions that simulate the high nitrogen availability typical of intensive cropping systems. Maize seedlings are especially sensitive to ammonium stress during early development, making this stage critical for assessing potential mitigation strategies.

Therefore, this study aimed to evaluate whether the role of Si in mitigating ammonium stress in maize seedlings is more related to the improvement in the conversion of N into dry matter, that is, to the increase in nitrogen use efficiency, rather than to the proline antioxidant production.

## 2. Results

### 2.1. Shoot Growth

**Figure 1:**
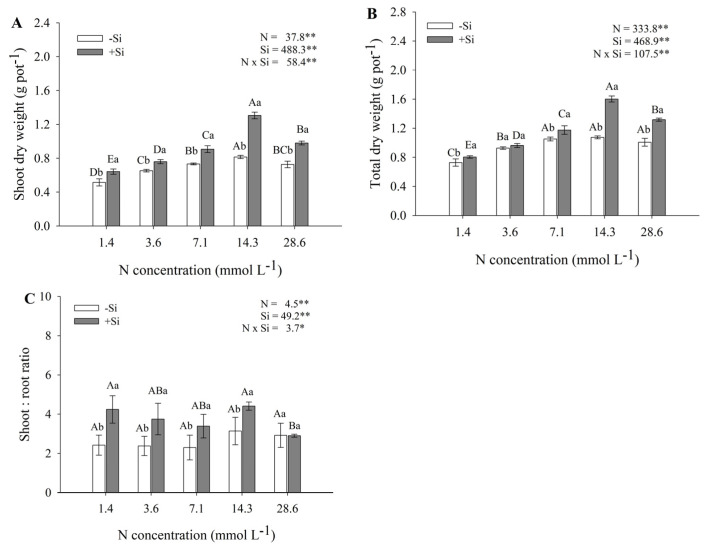
Shoot dry mass (**A**), whole-plant dry mass (**B**), and shoot-to-root ratio (**C**) of maize plants under different N–NH_4_^+^ concentrations, in the absence and presence of Si. Error bars represent the standard error of the mean (*n* = 4). Uppercase letters compare means among N–NH_4_^+^ concentrations, and lowercase letters compare means between Si treatments. Means followed by the same letter do not differ significantly according to Tukey’s test (*p* ≤ 0.05). *—significant difference at *p* < 0.05; **—highly significant difference at *p* < 0.01.

Increasing N–NH_4_^+^ concentrations in the nutrient solution promoted a significant increase in maize shoot growth. The greatest shoot and whole-plant biomass was observed at 14.3 mmol L^−1^ of N, both in the absence (0.80 and 1.0 g of dry mass) and in the presence of Si (1.3 and 1.6 g of dry mass, respectively). However, the stress caused by the highest N–NH_4_^+^ concentration (28.6 mmol L^−1^) inhibited shoot growth in plants grown without Si, resulting in reductions in dry mass (Figure 1A,B). Regardless of the N dose applied, the addition of Si resulted in higher shoot dry mass values compared with plants grown without the element. The greatest effect of silicon was observed at the higher ammonium concentrations (14.3 and 28.6 mmol L^−1^), at which plants exhibited the greatest biomass accumulation. The significant interaction between the factors (N × Si = 58.4 **) demonstrates that the plant response to ammonium depends on the presence of silicon. Additionally, an increase in the shoot-to-root ratio was observed in the presence of Si (Figure 1C).

**Figure 2:**
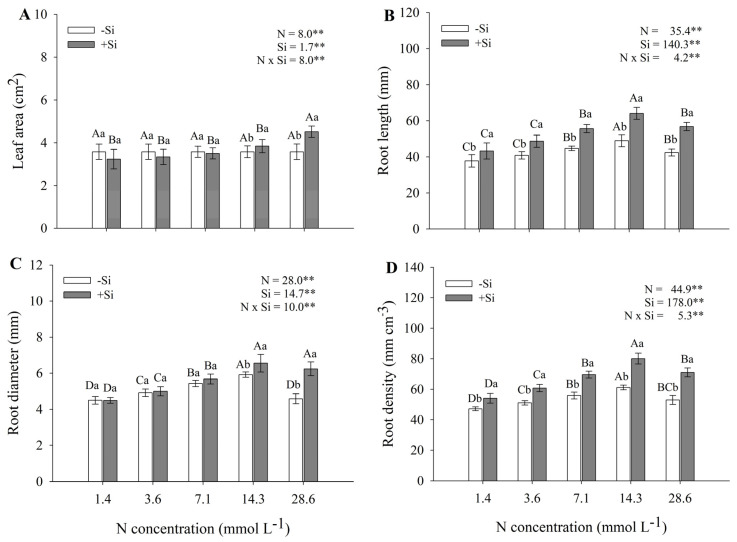
Leaf area (**A**), root length (**B**), root diameter (**C**), and root density (**D**) of maize seedlings grown under different N–NH_4_^+^ concentrations, in the absence and presence of silicon. Error bars represent the standard error of the mean (*n* = 4). Uppercase letters compare means among N–NH_4_^+^ concentrations, and lowercase letters compare means between Si treatments. Means followed by the same letter do not differ significantly according to Tukey’s test (*p* ≤ 0.05). **—highly significant difference at *p* < 0.01.

Leaf area, as well as root length, diameter, and density, showed a significant N × Si interaction, indicating that these factors do not act independently but mutually influence the plant response. Maize showed greater leaf area at N concentrations of 14.3 and 28.6 mmol L^−1^ when grown with Si, with values significantly higher than those observed in the treatments without Si at the same N concentrations (Figure 2A).

Root length, diameter, and density reached their highest values at the 14.3 mmol L^−1^ N concentration, with these parameters being significantly greater in plants grown in the presence of Si compared with those without Si application (Figure 2B–D). In the Si treatment, root length at this N concentration was 64.1 mm, representing a 48% increase relative to the lowest concentration (1.4 mmol L^−1^ N). Without Si, root length at the same N level was 48.9 mm—31% lower than in Si-treated plants.

The two highest N concentrations (14.3 and 28.6 mmol L^−1^ N) in the presence of Si promoted the greatest root diameter expansion, reaching 6.6 mm—approximately 46% greater than that observed at the lowest concentration (1.4 mmol L^−1^ N). In contrast, the maximum root diameter in the absence of Si occurred at 14.3 mmol L^−1^ N, reaching 5.9 mm. Overall, plants without Si exhibited root diameters 13% smaller than those of Si-supplemented plants.

The highest root density was observed in plants treated with Si at the N concentration of 14.3 mmol L^−1^, reaching 80.1 mm cm^−3^. In the absence of Si, the maximum root density was 13.9 mm cm^−3^, also observed at 14.3 mmol L^−1^ N, representing a 29% increase compared with the lowest N concentration. In addition, root length, diameter, and density decreased at the N concentration of 28.6 mmol L^−1^, differing statistically from the other treatments and between plants grown with and without Si, indicating the effects of N stress.

### 2.2. Accumulation and Efficiency Indices of N and Si Uptake and Utilization in Maize Plants

**Figure 3:**
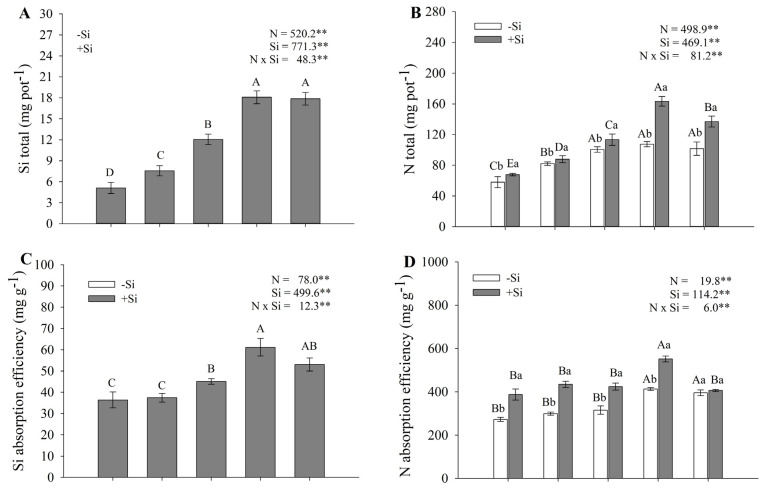

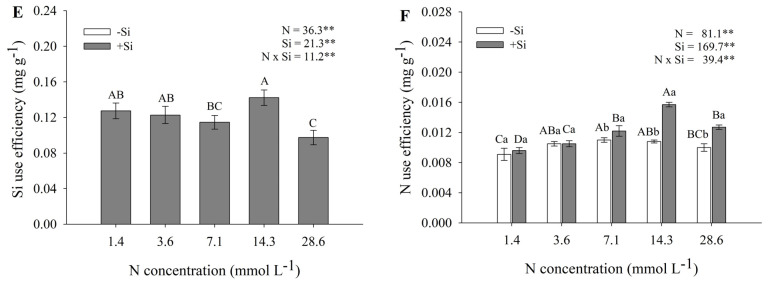
Accumulation of Si (**A**) and N (**B**), Si uptake efficiency index (**C**), N uptake efficiency index (**D**), Si utilization efficiency index (**E**), and N utilization efficiency index (**F**) in maize seedlings grown under different N-NH_4_^+^ concentrations, in the absence and presence of Si and were calculated as described in Section 4.3. Bars represent the standard error of the mean (*n* = 4). Uppercase letters compare means among N–NH_4_^+^ concentrations, and lowercase letters compare means among Si concentrations. Means followed by the same letter do not differ significantly according to Tukey’s test (*p* ≤ 0.05). **—highly significant difference at *p* < 0.01.

Statistical differences were also observed for Si and N accumulation, for the Si uptake efficiency index, for the N uptake efficiency index, for the Si utilization efficiency index, and for the N utilization efficiency index. Maize plants receiving Si accumulated more N and Si as N–NH_4_^+^ concentrations increased in the nutrient solution (Figure 3A,B). The highest Si uptake was observed in plants cultivated with Si supplementation, particularly at concentrations of 14.3 and 28.6 mmol L^−1^ of N, resulting in an increase of 13.0 mg of Si per pot compared to the treatment with 1.4 mmol L^−1^ of N. The presence of Si also significantly enhanced N accumulation, especially at the concentration of 14.3 mmol L^−1^ of N, where an accumulation of 87.7 mg of N per pot was observed, representing up to a 60% increase compared with the lowest concentration evaluated (1.4 mmol L^−1^) (Figure 3B). In contrast, in the absence of Si, no significant differences in N accumulation were observed among the three highest N concentrations.

The highest N and Si uptake and utilization efficiencies were recorded in the presence of Si. The combination of 14.3 mmol L^−1^ N and Si application produced the highest N uptake (551.6 mg pot^−1^) and utilization efficiency (0.0157 mg g^−1^ dry matter), corresponding to increases of 30–40% relative to 1.4 mmol L^−1^ N (Figure 3D,F). At 28.6 mmol L^−1^ N, N uptake decreased to values similar to those of the lowest N treatment, indicating a potential mitigating role of Si in preventing excessive or toxic N–NH_4_^+^ accumulation when nutrient availability is high. Without Si, N concentrations of 14.3 and 28.6 mmol L^−1^ yielded the highest N accumulation (413.2 and 395.7 mg pot^−1^), representing increases of up to 34% compared with 1.4 mmol L^−1^ N. Maximum N utilization efficiency was observed at 7.1 mmol L^−1^ N, though it did not differ statistically from 3.6 and 14.3 mmol L^−1^ N in the –Si treatment (Figure 3F).

Maize showed greater efficiency in Si uptake (Figure 3C) and Si conversion into dry matter (Figure 3E) when grown at 14.3 mmol L^−1^ N. At this concentration, Si uptake efficiency at 61.2 mg g^−1^. However, at higher N supply, Si utilization efficiency decreased to the lowest values among the treatments evaluated, differing statistically from all other ammonium levels.

### 2.3. Relative Chlorophyll Content (SPAD), Free Proline in Roots, and Nitrate Reductase Activity in Maize

**Figure 4:**
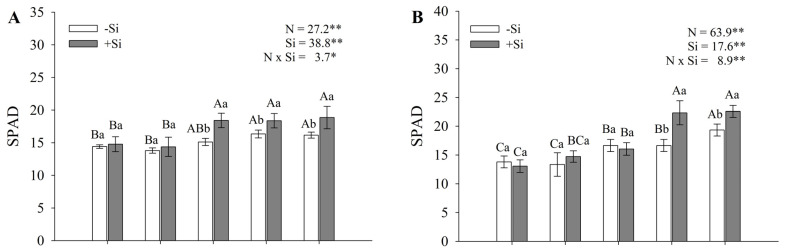

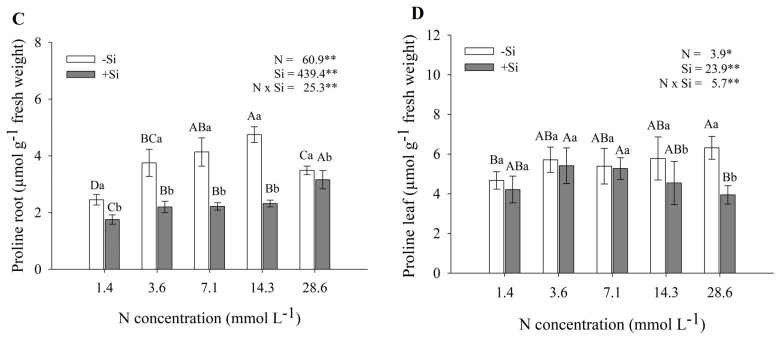
SPAD index evaluated at 10 DAT (**A**) and 20 DAT (**B**), and proline concentration in roots (**C**) and shoots (**D**) of maize seedlings grown under different N-NH_4_^+^ concentrations, in the absence and presence of Si. Bars represent the standard error of the mean (*n* = 4). Uppercase letters compare means among N–NH_4_^+^ concentrations, and lowercase letters compare means among Si concentrations. Means followed by the same letter do not differ significantly according to Tukey’s test (*p* ≤ 0.05). *—significant difference at *p* < 0.05. **—highly significant difference at *p* < 0.01.

SPAD values differed significantly according to N, Si, and their interaction at both evaluation times. SPAD indices increased with higher N concentrations, both in the presence and absence of Si, at both evaluation times. However, plants grown in the presence of Si showed significantly higher SPAD indices, especially at the higher N concentrations. The differences were more pronounced in the treatments with 14.3 and 28.6 mmol L^−1^ of N, with increases of 24% and 42% in SPAD values at 10 DAT (Figure 4A) and 20 DAT (Figure 4B), respectively, compared with treatments without Si. In the absence of Si, the increase in N concentrations also resulted in higher SPAD indices, with values ranging from 17.0 to 19.3 in leaves evaluated at the highest N concentration, regardless of the evaluation time.

The highest free proline contents were observed in seedlings grown in the absence of Si, reaching 4.8 µmol g^−1^ in roots (Figure 4C) and 6.3 µmol g^−1^ in shoots (Figure 4D), at N concentrations of 14.3 and 28.6 mmol L^−1^, respectively. Across all N concentrations, Si presence reduced root proline accumulation significantly; in shoots, reductions were significant only at the two highest N levels, indicating that Si mitigates stress induced by elevated N–NH_4_^+^.

In Si-treated plants, proline accumulation in shoots increased to 5.4 µmol g^−1^ at 7.1 mmol L^−1^ N. Beyond this point, a marked decrease in proline content was observed, so that at the highest N concentration (28.6 mmol L^−1^), plants showed 3.9 µmol g^−1^ of proline, a value lower than that recorded at the lowest N concentration. In contrast, in the absence of Si, proline contents in shoots increased by 26% compared with the lowest N concentration evaluated and were up to 40% higher than those observed in plants grown with Si, highlighting the role of silicon in mitigating stress induced by excess N-NH_4_^+^.

**Figure 5:**
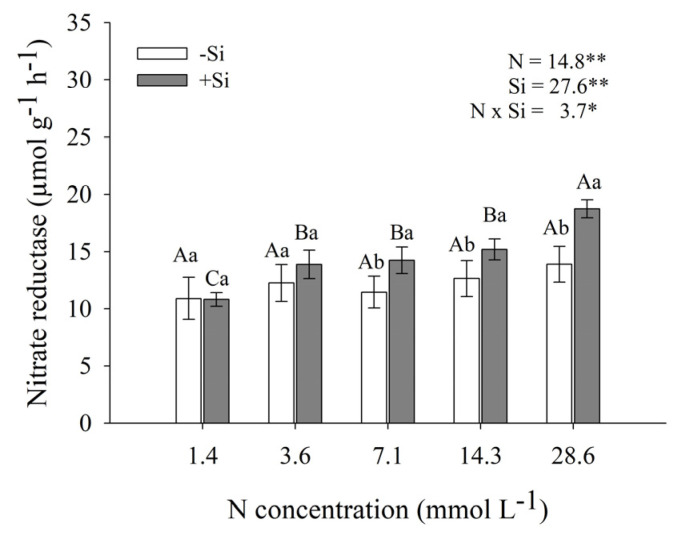
Nitrate reductase activity in maize shoots grown under different N-NH_4_^+^ concentrations, in the absence and presence of silicon (Si). Bars represent the standard error of the mean (*n* = 4). Uppercase letters compare means among N–NH_4_^+^ concentrations, and lowercase letters compare means among Si concentrations. Means followed by the same letter do not differ significantly according to Tukey’s test (*p* ≤ 0.05). *—significant difference at *p* < 0.05; **—highly significant difference at *p* < 0.01.

The presence of Si stimulated nitrate reductase activity in maize shoots, especially at the higher N concentrations in the nutrient solution. The peak enzyme activity was observed at 28.6 mmol L^−1^ of N, reaching 18.7 µmol g^−1^ h^−1^, representing a 47% increase compared with the lowest concentration evaluated (1.4 mmol L^−1^ of N) (Figure 5). With the exception of the 1.4 and 3.6 mmol L^−1^ N treatments, all other Si-supplemented treatments showed significantly higher activity than that observed in plants grown without this element. In contrast, in plants grown in the absence of Si, increasing N concentrations did not result in significant variations in nitrate reductase activity, as indicated by statistical analysis, suggesting a role for Si in modulating enzyme activity under different N availability conditions.

### 2.4. Pearson Correlation Analysis

**Figure 6:**
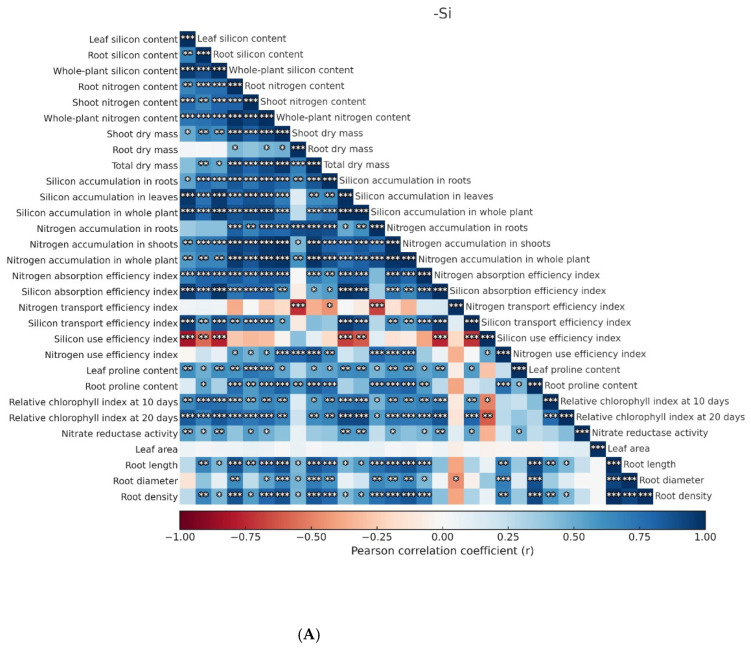

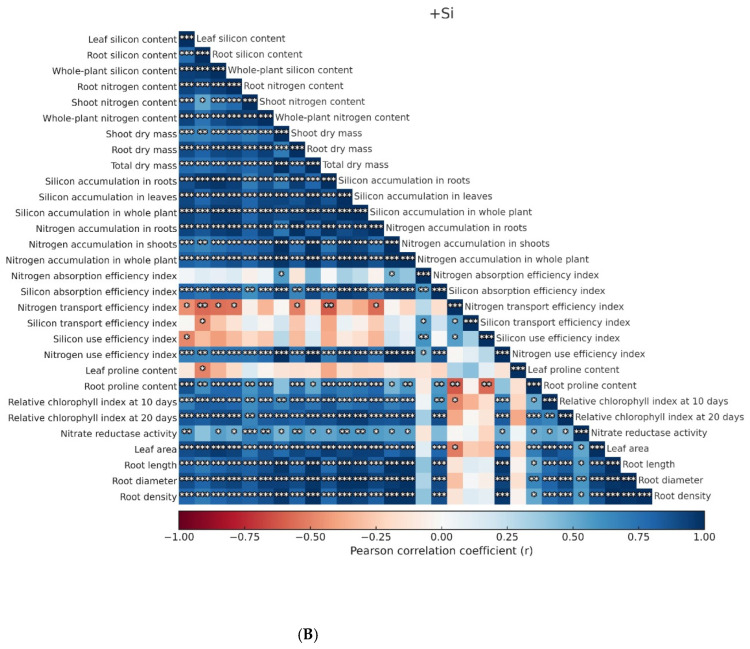
Pearson’s correlation network of growth, nutritional, biochemical, and photosynthetic variables in maize seedlings grown under different N–NH_4_^+^ concentrations, without (**A**) or with (**B**) silicon (Si). *—significant difference at *p* < 0.05; **—highly significant difference at *p* < 0.01; ***—*p* < 0.001.

A Pearson correlation plot was constructed to analyze the relationships among the different parameters evaluated—including growth traits, nutritional, biochemical, and photosynthetic aspects—in maize plants (Figure 6). The results indicated that, in the absence of silicon (Si), the total nitrogen utilization efficiency index showed a negative correlation with Si accumulation in roots and Si concentration in shoots. In addition, the nitrogen utilization efficiency index displayed negative correlations with several variables: total Si accumulation in the plant, N accumulation in shoots, chlorophyll content estimated at 20 days after transplanting (SPAD at 20 DAT), Si uptake efficiency index, Si concentration in roots, chlorophyll content estimated at 10 days after transplanting (SPAD at 10 DAT), and Si utilization efficiency index. Leaf area was the only variable that did not show a significant correlation with the others in the absence of Si in the nutrient solution.

The addition of N and Si to the nutrient solution promoted an increase in total Si accumulation in the plant (Figure 3), which showed a positive correlation with several maize growth parameters, including root dry weight, shoot dry weight, and total plant dry weight, as well as leaf area, root diameter, root density, and root length (Figure 6B). This growth enhancement may also be associated with the positive correlation between Si accumulation in whole plant and chlorophyll contents estimated at 10 (SPAD at 10 DAT) and 20 days after transplanting (SPAD at 20 DAT). Silicon is known to protect photosynthetic pigments and reduce structural damage to chloroplasts, as well as to be involved in upregulating genes related to photosynthesis [23]. Thus, the increase in chlorophyll content may result in higher photosynthetic efficiency and, consequently, greater carbon conversion into dry matter. The higher plant growth can also be attributed to the positive correlation between Si accumulation in whole plant and nitrate reductase activity, a key enzyme in N assimilation that plays an essential role in amino acid and protein synthesis. These compounds, in turn, are fundamental for structural and physiological functions in plant cells, including cell division and expansion, as well as the development of new tissues. Furthermore, Si accumulation in whole plant in seedlings grown with Si was positively correlated with proline contents in leaves (Free proline in shoots) and roots (Free proline in roots).

## 3. Discussion

Our results provide new insights into the role of Si in maize subjected to ammonium nutrition. In this study, maize was cultivated under different NH_4_^+^ concentrations, in the presence and absence of Si. The main conclusions were that (a) maize is tolerant to high NH_4_^+^ concentrations, (b) Si accumulation varied according to the NH_4_^+^ concentration in the nutrient solution, and (c) Si mitigates NH_4_^+^ stress in maize by increasing nitrogen use efficiency. Additionally, our findings help clarify limitations present in the literature, particularly in the study by [24], which evaluated the effects of Si under conditions of high ammonium availability (30 and 60 mmol L^−1^). Although those authors reported a partial reduction in stress symptoms, their experimental design presented important methodological restrictions that hinder the interpretation of the physiological mechanisms involved. In this context, our study advances the understanding of this interaction by employing a broader and more realistic range of NH_4_^+^ concentrations, together with an adequate Si dose, allowing for the identification of more consistent physiological responses and the elucidation of mechanisms directly related to nitrogen uptake, assimilation, and utilization. Thus, this study fills gaps left by previous research and provides more robust evidence regarding the interaction between Si and N–NH_4_^+^ in maize seedlings. These conclusions are discussed in detail below.

The compiled data presented by [5] indicated that maize is among the plant species considered tolerant to NH_4_^+^. This observation was corroborated in the present study, since the points of maximum plant growth (Figure 1 and Figure 2), as well as N accumulation, uptake efficiency, and utilization efficiency (Figure 3), were reached between 14.3 and 28.6 mmol L^−1^ of NH_4_^+^. These concentrations are classified as high, as previously reported in review studies by [25,26] for other plant species. Therefore, our results reinforce the evidence that maize is indeed tolerant to elevated NH_4_^+^ supply.

The addition of Si increased root length, diameter, and density, as well as leaf area and plant dry matter, when compared to plants grown without Si supplementation, highlighting its beneficial effects under NH_4_^+^ stress. Even at 28.6 mmol L^−1^ NH_4_^+^—a concentration considered highly toxic—Si supplementation promoted better plant development compared with treatments without this element, although growth was reduced compared to 14.8 mmol L^−1^ NH_4_^+^ [27], evaluating the mitigation of ammonium stress by Si in tomato plants, emphasized that the beneficial effects of Si depend on the ammonium concentration in the nutrient solution. Similarly, Ref. [26] reported that the enhanced plant growth observed under Si supply can be attributed to its role in stimulating carbon accumulation, which in turn improves nitrogen uptake and supports plant development, even under excessive NH_4_^+^ availability. Moreover, Si deposition in cell walls has been associated with increased protection against cellular damage induced by NH_4_^+^ stress. In addition, the increase in the shoot-to-root ratio observed in the presence of Si may indicate a shift in carbon allocation toward the aerial parts of the plant (Figure 1C). Supporting this interpretation [28], found that Si addition significantly decreased structural carbon contents and simultaneously improved photosynthetic performance and biomass accumulation. This indicates that the carbon spared from structural functions may have been reallocated to metabolic and growth processes—a concept that aligns with our findings of a higher shoot/root ratio under Si supply.

The accumulation of Si and N in the maize shoots varied according to both NH_4_^+^ and Si concentrations in the nutrient solution. Based on the classification proposed by [29], maize plants grown under 1.4 and 3.6 mmol L^−1^ NH_4_^+^ accumulated Si at rates that categorize them as intermediate accumulators. However, at 14.3 and 28.6 mmol L^−1^ NH_4_^+^, where shoot and root growth were maximized, plants absorbed Si at rates consistent with Si accumulators, reaching values of 18.0 and 17.8 g plant^−1^, respectively (Figure 3A). It is noteworthy that higher NH_4_^+^ concentrations were consistently associated with greater Si accumulation. Supporting this, Ref. [30] proposed that root H^+^ exudation, resulting from the nitrification of NH_4_^+^ and subsequent rhizosphere acidification in sugarcane, can enhance Si uptake from external sources. In the present study, the silicic acid (H_2_SiO_3_) used as the Si source may have reacted with root-released H^+^, forming H_4_SiO_4_, the more readily absorbed form of this element by plants.

The highest N accumulation was observed in treatments supplied with Si, with the maximum occurring at 14.3 mmol L^−1^ NH_4_^+^. Previous studies have highlighted the role of Si in promoting plant growth and improving nutrient use efficiency, including N [31,32]. According to [24], this effect can be attributed to the interaction between Si and N metabolism, as Si enhances photosynthesis, stabilizes chloroplast structure, and increases the activity of enzymes responsible for N reduction and assimilation. In the absence of Si, N accumulation was lower than in Si-supplied plants, which can be explained by the higher N use efficiency observed under Si supplementation (Figure 3F), resulting in greater biomass production (Figure 1). The fact that maximum N accumulation occurred at 14.3 mmol L^−1^ NH_4_^+^ rather than at 28.6 mmol L^−1^, even in the presence of Si, may be related to root damage at the higher NH_4_^+^ concentration (Figure 1C). Excess ammonium is known to impair root growth and cause necrosis [24], even in tolerant species, thereby reducing N uptake. Another possible explanation is the excessive accumulation of NH_4_^+^ in chloroplasts, which may disrupt the activity of the glutamine synthetase–glutamate synthase (GS–GOGAT) enzyme complex, a key pathway for N assimilation in plants [10]. Excessive accumulation of ammonium in the chloroplasts can induce various disturbances, thereby exacerbating ammonium stress—a phenomenon well documented in the literature. These disturbances are associated with the direct inhibition of enzymatic activity (GS/GOGAT), the uncoupling of photosynthesis and dissipation of the pH gradient (proton electrochemical gradient) required for ATP synthesis, an imbalance in C and N metabolism, reduced Rubisco activity, and damage to thylakoid membranes.

Both Si and N uptake and utilization efficiencies increased up to 14.3 mmol L^−1^ NH_4_^+^ (Figure 3). Si has been previously reported to promote plant growth and enhance nutrient use efficiency, including N [31,32]. Si application improves N utilization efficiency, likely through the upregulation of enzymes involved in N assimilation [10,21,33]. In contrast, at 28.6 mmol L^−1^ NH_4_^+^, the efficiencies of both elements declined, irrespective of Si supply, due to the toxicity of this cation at elevated concentrations in plant tissues [27].

SPAD values increased with rising NH_4_^+^ concentrations and were further enhanced by Si supply (Figure 4A,B). Similar findings were reported for maize by [15], who attributed this effect to the strong correlation between chlorophyll and leaf N content, as 50–70% of leaf N is allocated to chloroplast-associated enzymes. In addition, Si deposition in epidermal cells promotes more erect leaves, improving light interception, while also protecting the photosynthetic apparatus against the detrimental effects of ammonium stress [34,35].

The results showed that free proline accumulation in leaves and roots was stimulated by NH_4_^+^ supply, whereas Si addition reduced this physiological parameter (Figure 4C,D). Proline is a multifunctional amino acid with protective roles, including the mitigation of abiotic stresses such as excess N [36]. Si has also been reported to confer protection against abiotic stress [37]. However [38], observed in alfalfa that Si supplementation in the nutrient solution decreased free proline content, which was attributed to enhanced proline oxidation.

It is important to emphasize that ammonium stress in this experiment reached levels considered moderate. The use of the highest ammonium-N concentration, compared with the lower concentrations of this nutrient, caused damage to the root system of plants that did not receive Si, significantly reducing different root variables, such as root diameter, length, and density (Figure 2B–D). This is due to oxidative stress induced in the plant root, which can be confirmed by the significant decrease in proline content (Figure 4C). These morphological damages to the root, together with the weakening of the plant’s defense system, led to a reduction in plant dry mass production (Figure 1A) when comparing the highest ammonium-N concentration with the optimal concentration of this nutrient (14.3 mmol L^−1^) in plants that did not receive Si. Therefore, this root damage caused by the use of the highest ammonium concentration in the nutrient solution was sufficient to affect physiological aspects responsible for the conversion of ammonium into shoot dry mass.

Another variable influenced by Si was NR activity (Figure 5). This enzyme plays a key role in N reduction and assimilation by catalyzing the conversion of nitrate to nitrite, which is subsequently reduced to ammonium by NiR [39]. Ammonium is then incorporated into amino acids through the GS–GOGAT pathway [40]. Thus, increased NR activity may enhance the availability of amino acids and proteins, favoring dry matter accumulation. In the present study, the stimulation of NR activity by Si application promoted maize growth and SPAD values (Figure 2, Figure 4A,B, and Figure 5), mitigating the negative effects of excess NH_4_^+^. These findings are consistent with previous reports highlighting the role of Si in regulating N metabolism and NR activity [41,42].

This study revealed strong correlations between growth and physiological parameters in maize seedlings (Figure 6), underscoring the substantial improvements promoted by Si. Beyond acting as an effective modulator under challenging nutritional conditions, Si enhanced plant resilience to abiotic stress, thereby increasing adaptive capacity [35]. These findings not only advance our understanding of the agronomic benefits of Si but also emphasize its pivotal role in sustaining plant growth under adverse nutritional scenarios. Importantly, the acidic stress derived from excessive ammonium (NH_4_^+^) assimilation is considered the main driver of NH_4_^+^ stress. Hence, further molecular investigations into the interaction between Si and H^+^ ions under high NH_4_^+^ supply are warranted to provide deeper insights into the mechanisms by which Si mitigates ammonium stress.

## 4. Materials and Methods

The study was conducted at the School of Agricultural and Veterinary Sciences, São Paulo State University (UNESP), Jaboticabal campus, São Paulo, in a plant growth room under controlled conditions of light, photoperiod, and relative air humidity. Seeds of the maize hybrid AG 1051 were surface-sterilized with a 5% sodium hypochlorite (NaClO) solution for five minutes, rinsed with deionized water, and subsequently germinated in plastic trays containing sterile sand, maintained at 25 °C in a germination chamber.

Four days after sowing, upon the emergence of the second leaf pair, two uniform seedlings were transplanted into plastic pots containing 0.8 L of [43] nutrient solution, initially at one-quarter ionic strength for three days, followed by three days at three-quarters ionic strength to allow adaptation to the hydroponic system. After this period, the seedlings were exposed to full-strength [43] nutrient solution, modified according to the NH_4_^+^ concentrations established for the experiment. At this stage, two nutrient solutions were prepared: one containing 1.8 mmol L^−1^ of silicon (Si) and another devoid of this element.

Deionized water was used for the preparation of the nutrient solutions, which were continuously aerated throughout the experiment. Based on the formulation proposed by [42], stock solutions were prepared for the treatments using the following salts: KH_2_PO_4_, NH_4_H_2_PO_4_, KNO_3_, MgSO_4_·7H_2_O, Ca(NO_3_)_2_·5H_2_O, KCl, NH_4_NO_3_, CaCl_2_·2H_2_O, MnCl_2_·4H_2_O, CuCl_2_, H_2_MoO_4_·H_2_O, H_3_BO_3_, ZnCl_2_, and Fe-EDDHA. Water lost through evapotranspiration was replenished directly in the pots with deionized water. The pH of the solutions was monitored every 12 h and maintained at 5.8 ± 0.1 through the addition of 0.5 mol L^−1^ NaOH or HCl as required. Nutrient solution replacement was performed whenever a 30% reduction in the initial electrical conductivity was observed. Plants remained in the growth chamber for 22 days under controlled conditions of temperature (25 ± 1 °C), relative humidity (50 ± 5%), light intensity (450 μmol m^−2^ s^−1^), and a 12-h photoperiod (light/dark).

### 4.1. Experimental Design

The experiment was conducted in a completely randomized design, arranged in a 5 × 2 factorial scheme with four replications. Each experimental unit consisted of a pot containing two maize plants. The treatments consisted of five nitrogen concentrations (1.4, 3.6, 7.1, 14.3, and 28.6 mmol L^−1^), applied in the presence or absence of silicon (1.8 mmol L^−1^ Si). The nitrogen source was a combination of nitrate and ammonium in a N-NO_3_^−^:N-NH_4_^+^ ratio of 4:5. Silicon was supplied as monosilicic acid (H_2_SiO_3_) (Sigma-Aldrich, St. Louis, MO, USA), selected based on preliminary tests that demonstrated its superior solubility in the nutrient solution compared with other Si sources.

### 4.2. Determination of Root Morphology and Maize Plant Growth

For root morphology assessment, root samples were carefully collected at harvest, 22 days after planting, and stained with methylene blue (10 mg mL^−1^) for approximately two minutes. Subsequently, the roots were rinsed under running water and placed in a tray containing water, where they were scanned for morphological analysis. Following the method described by [44], the Delta-T Scan image analysis software, model DTS-COM110 (Delta-T Devices, Cambridge, UK), was used to quantify root length (mm), diameter (mm), area (cm^2^), and root density (mm cm^−3^) of maize plants.

Subsequently, the plants were separated into shoots and roots. The roots were washed under running water to remove residues. The shoots were sequentially washed with running water, 0.1% neutral detergent solution, 0.3% hydrochloric acid solution, and finally rinsed with deionized water. The plant material was then dried in a forced-air oven at 65 ± 5 °C until a constant weight was achieved. After drying, the dry mass of the shoots and roots was determined.

### 4.3. Determination of N and Si Accumulation in Plant Tissue

Nitrogen concentrations in leaves and roots were determined from 0.3 g of dry mass through digestion with concentrated hydrochloric acid, followed by quantification via titration with 0.1 M HCl, according to the Kjeldahl method described by [45]. For silicon determination, 0.1 g of dry leaf and root tissue was digested in polyethylene tubes using 50% NaOH. Subsequently, the samples were treated with NH_4_F prior to Si quantification by molybdenum blue colorimetry, as described by [46]. The accumulation of each element was calculated by multiplying the measured concentration by the respective dry mass of roots and shoots.

### 4.4. Nutrient Uptake and Use Efficiency Indices

Based on the dry mass and N and Si contents in the whole plant, shoots, and roots, the following indices were calculated: (i) uptake efficiency (UE), determined as the total accumulation of the element in the plant (A_plant_) divided by root dry mass (DM_root_) − UE = A_plant_/DM_root_; and (ii) utilization efficiency (UtE), defined as the square of the total dry mass (DM_total_^2^) divided by the total accumulation of the element in the plant (A_plant_) − UtE = DM_total_^2^/A_plant_, according to [47].

### 4.5. Proline and Leaf Greenness Index

Proline concentration was determined in leaf tissues (200 mg) following the method described by [48]. Samples were homogenized in 3% (*w*/*v*) sulfosalicylic acid, and the filtered homogenate was reacted with acidic ninhydrin at 100 °C for 1 h. The reaction mixture was then extracted with toluene, and the absorbance of the samples was measured at a wavelength of 520 nm.

At 10 and 20 days after treatment application, an indirect measurement of chlorophyll content (leaf greenness index—SPAD) was performed on two plants per pot, using the first fully expanded leaf from the apex of the plant. Measurements were taken with a Chlorophyll Content Meter (CCM 200; Opti-Sciences^®^, Hudson, NH, USA).

### 4.6. Nitrate Reductase (E.C. 1.6.6.1)

Nitrate reductase activity was determined according to the method described by [49]. Twenty-one days after treatment application, 1 g fresh weight samples were collected from the middle region of maize leaves. The samples were placed in dark flasks containing 10 mL of incubation medium, composed of 6.0 mL of 400 mM phosphate buffer, 6.0 mL of 200 mM KNO_3_, 6.0 mL of 4% (*v*/*v*) n-propanol, 2.4 mL of 0.1% (*v*/*v*) Triton X-100, and 3.6 mL of deionized water, with the pH adjusted to 7.2.

The flasks were placed in a desiccator and subjected to vacuum (60 cm Hg) for one minute, followed by reintroduction of air. This procedure was repeated three times. Subsequently, the flasks were incubated in a water bath at 30 °C in the dark for 90 min. The reaction was stopped by the addition of 1 mL of 1% sulfanilamide solution. Due to nitrite formation by nitrate reductase activity, 0.5 mL of the incubation medium was withdrawn for nitrite quantification, following [50]. To this aliquot, 0.5 mL of 1% sulfanilamide solution and 0.5 mL of 0.02% N-1-naphthylethylenediamine hydrochloride solution were added. The final solution was allowed to stand for 20 min and then diluted with deionized water to a final volume of 4.0 mL. Sample absorbance was measured at 540 nm, as described by [51].

### 4.7. Statistical Analysis

Data variance was analyzed using the F-test (α ≤ 0.05), and treatment means that differed significantly were identified by Tukey’s test at *p* ≤ 0.05. All analyses were performed using AgroEstat software 1.0 version [52].

## 5. Conclusions

Silicon supplementation alleviated ammonium stress in maize seedlings, promoting growth, improving nitrogen use efficiency, and enhancing physiological performance. These benefits highlight the potential of Si as a practical strategy to increase crop resilience and productivity under challenging nutrient conditions.

## Data Availability

The raw data supporting the conclusions of this article will be made available by the authors on request.
